# Butyric Acid Supplementation Reduces Changes in the Taxonomic and Functional Composition of Gut Microbiota Caused by *H. pylori* Eradication Therapy

**DOI:** 10.3390/microorganisms12020319

**Published:** 2024-02-03

**Authors:** Sayar Abdulkhakov, Maria Markelova, Dilyara Safina, Maria Siniagina, Dilyara Khusnutdinova, Rustam Abdulkhakov, Tatiana Grigoryeva

**Affiliations:** 1 Institute of Fundamental Medicine and Biology, Kazan (Volga Region) Federal University, 420008 Kazan, Russia; mimarkelova@gmail.com (M.M.); diliarik@yandex.ru (D.S.); marias25@mail.ru (M.S.); dilyahusn@gmail.com (D.K.); tatabio@inbox.ru (T.G.); 2 Department of Outpatient Therapy and General Medical Practice, Kazan State Medical University, 420012 Kazan, Russia; 3 Department of Hospital Therapy, Kazan State Medical University, 420012 Kazan, Russia; rustemabdul@mail.ru

**Keywords:** gut microbiota, *H. pylori*, metagenome, eradication therapy, shotgun sequencing, butyric acid+inulin (Zacofalk)

## Abstract

*H. pylori* eradication therapy leads to significant changes in the gut microbiome, including influence on the gut microbiome’s functional potential. Probiotics are one of the most studied potential methods for reducing the microbiota-related consequences of antibiotics. However, the beneficial effects of probiotics are still under discussion. In addition, there are some concerns about the safety of probiotics, emphasizing the need for research of other therapeutic interventions. The aim of our study was to evaluate the influence of butyric acid+inulin supplements on gut microbiota changes (the gut microbiota composition, abundance of metabolic pathways, and gut resistome) caused by *H. pylori* eradication therapy. Materials and methods. Twenty two *H. pylori*-positive patients, aged 19 to 64 years, were enrolled in the study and randomized into two treatment groups, as follows: (1) ECAB-14 (n = 11), with esomeprazole 20 mg, clarithromycin 500 mg, amoxicillin 1000 mg, and bismuthate tripotassium dicitrate 240 mg, twice daily, per os, for 14 days, and (2), ECAB-Z-14 (n = 11), with esomeprazole 20 mg, clarithromycin 500 mg, amoxicillin 1000 mg, and bismuthate tripotassium dicitrate 240 mg, twice daily, along with butyric acid+inulin (Zacofalk), two tablets daily, each containing 250 mg of butyric acid, and 250 mg of inulin, per os, for 14 days. Fecal samples were collected from each subject prior to eradication therapy (time point I), after the end of eradication therapy (time point II), and a month after the end of eradication therapy (time point III). The total DNA from the fecal samples was isolated for whole genome sequencing using the Illumina NextSeq 500 platform. Qualitative and quantitative changes in gut microbiota were assessed, including alpha and beta diversity, functional potential and antibiotic resistance gene profiling. Results. Gut microbiota alpha diversity significantly decreased compared with the baseline immediately after eradication therapy in both treatment groups (ECAB-14 and ECAB-Z-14). This diversity reached its baseline in the ECAB-Z-14 treatment group a month after the end of eradication therapy. However, in the ECAB-14 treatment arm, a reduction in the Shannon index was observed up to a month after the end of *H. pylori* eradication therapy. Fewer alterations in the gut microbiota functional potential were observed in the ECAB-Z-14 treatment group. The abundance of genes responsible for the metabolic pathway associated with butyrate production decreased only in the ECAB-14 treatment group. The prevalence of antibiotic-resistant genes in the gut microbiota increased significantly in both treatment groups by the end of treatment. However, more severe alterations were noted in the ECAB-14 treatment group. Conclusions. *H. pylori* eradication therapy leads to taxonomic changes, a reduction in the alpha diversity index, and alterations in the functional potential of the gut microbiota and gut resistome. Taking butyric acid+inulin supplements during *H. pylori* eradication therapy could help maintain the gut microbiota in its initial state and facilitate its recovery after *H. pylori* eradication.

## 1. Introduction

There is a clear need for *H. pylori* eradication in patients with gastrointestinal diseases, such as peptic ulcer disease, atrophic gastritis, and gastric MALToma. Moreover, it is now widely accepted that *H. pylori*-associated gastritis is an infectious disease, regardless of symptoms and complications, and that *H. pylori* eradication can cure gastritis and improve future prognosis [1,2,3,4]. In addition, the International Agency for Research on Cancer (IACR) of the World Health Organization classified *H. pylori* as a Group 1 carcinogen [5], considering the presence of *H. pylori* as a risk factor for gastric adenocarcinoma [6,7] and *H. pylori*-associated gastritis as a precancerous lesion [8]. Successful *H. pylori* eradication may not only mitigate the risk of gastric cancer, but also promote atrophy involution of both the gastric corpus and the antrum mucosa in particular cases [1,9]. However, intestinal metaplasia is considered to be irreversible. Thus, there is presently significant evidence in favor of eradication therapy in *H. pylori*-positive patients to prevent gastric cancer, regardless of the clinical manifestations of the infection.

Antibiotic therapy, including therapy aimed at eradicating *H. pylori*, results in changes to the intestinal microbiota taxonomic composition and alpha diversity, as well as an abundance of metabolic pathways. Such therapy has a number of short- and long-term side effects, some of them caused by the negative influence of antibiotics on gut microbiota. These effects are largely due to a reduction in the abundance of short chain fatty acid (SCFA)-producing bacteria [1,10,11,12,13,14,15,16,17,18].

Therefore, potential methods for reducing these microbiota-related consequences are needed to ensure the safety of *H. pylori* eradication therapy.

Several studies have shown that probiotic supplementation could improve eradication rates, and reduce antibiotic-caused side effects related to microbiota changes, such as diarrhea, etc. [19,20,21]. Less-pronounced changes in microbial diversity and gut microbiota composition have been noted in cases of probiotic supplementation compared with therapies that do not add probiotics, but some results of those studies are inconsistent and contain data that only concern specific bacterial species [16,22,23,24,25,26]. Moreover, along with the generally observed beneficial effects of probiotic treatment in maintaining the gut microbiome and reducing antibiotic-induced adverse effects, the safety profiles of probiotic supplements remain a topic of discussion [27]. Based on these data, researchers have some concerns about the safety of probiotic therapy and they maintain that other, safer therapeutic interventions should be considered. Such proposed interventions include metabiotics and prebiotics.

Metabiotics refer to the metabolites of probiotic microorganisms, and they have advantages over traditional probiotics due to their known chemical structures and more pronounced positive impacts [28]. Butyrate is the most commonly studied SCFA, and it acts as a source of energy for both the gut microbiota and colonocytes, as well as a modulator for various metabolic activities, including the immunity of host cells [29]. Numerous in vivo and in vitro studies have further shown that SCFAs regulate inflammatory responses and are responsible for repairing intestinal barriers in the gut [30,31,32,33,34,35]. The benefits of most intestinal beneficial bacteria are also mediated by SCFAs, particularly butyric acid [29,36,37].

Prebiotics are believed to specifically stimulate the growth or activity of a particular number of commensal bacteria. However, the corresponding mechanism of action is difficult to predict as it depends on numerous factors, including the individual composition of gut microbiota [38].

The presently known prebiotic properties of inulin are known, due to the substance’s stimulation effects on *Bifidobacterium* and *Lactobacillus*, and they have been described through both in vitro and in vivo assessments in different laboratories [39,40,41]. Moreover, even synbiotics containing *Bifidobacterium lactis* and *Lactobacillus rhammnosus*, when enriched with inulin, were shown to regulate the intestinal microenvironment more effectively than single components [42,43]. This improved regulation is due to inulin fermentation, resulting in a significantly greater ratio of Lactobacillus and Bifidobacteria to Enterobacteria strains and an elevated butyrate concentration [44]. It is believed that elevated butyrate production can improve gut health by creating a more acidic environment, thereby increasing resistance to the colonization of pathogens [45]. Consequently, it was suggested that inulin supplements may contribute to overcoming the intestinal microbiota disturbances caused by antibacterial therapy.

Supplementing *H. pylori* eradication therapy with butyric acid+inulin was shown to reduce side effects related to antibiotics, thereby increasing compliance, and subsequently, the *H. pylori* eradication rate [19,46]. However, there are no sufficient data concerning the influence of such supplements on the gut microbiota. With this in mind, we hypothesized that supplementing *H. pylori* eradication therapy with a butyric acid+inulin combination could diminish the microbiota changes caused by antibiotics.

Current technologies allow one to assess the bacterial diversity and taxonomic or functional composition of human-associated microbiomes, as well as identify changes in responses to a specific supplementation. Our study sought to evaluate the effects of *H. pylori* eradication therapy on the gut microbiota (microbiota composition, the abundance of the most important metabolic pathways, and the prevalence of gut microbiota antibiotic-resistance genes), as well as the influence of butyric acid+inulin supplements on gut microbiota changes that occur during the eradication therapy.

## 2. Materials and Methods

### 2.1. Study Design

This prospective study was approved by the Local Ethics Committee of Kazan Federal University (protocol No. 1 dated 23 February 2015). Written informed consent was obtained from all included subjects visiting the outpatient gastroenterology unit of Kazan Federal University Hospital and Kazan State Medical University Out-patient Clinic (Kazan, Russian Federation).

Patients were selected for the study according to the following inclusion/exclusion criteria.

Inclusion Criteria:

(1) Patients of both sexes, aged 18 to 65;
(2) Upper gastrointestinal endoscopy and *H. pylori* detection with at least one method performed within one month prior to enrollment into the study;
(3) Signed written informed consent form;
(4) Informed consent to comply with the same dietary and cooking procedures throughout the study period (all the patients were requested to fill in the questionnaire concerning their daily meals throughout the study period).

Exclusion Criteria:

(1) Endoscopy-confirmed gastric polyps or cancer/malignancy;
(2) A history of concomitant diseases and conditions that might significantly affect the gut microbiota, such as the following:
  (a) Inflammatory bowel diseases;
  (b) Malabsorption syndrome associated with a documented disease of the small intestine, pancreas, etc.;
  (c) Cancers in any location;
  (d) Prior gastrointestinal surgeries (except for appendectomy);
  (e) The use of some drugs (immunosuppressive agents, cytostatics, steroids, antibiotics, and pre- and probiotics) within 3 months prior to enrollment into the study;
  (f) Functional bowel disorders, celiac disease, allergies, type 1 and type 2 diabetes, metabolic syndrome, non-alcoholic fatty liver disease, psychiatric disorders, etc., which, in the investigator’s view, may cause changes in the intestinal microbiota’s composition.
(3) The use of bismuth-containing drugs (Bismuthate tripotassium dicitrate) for 4 weeks, and proton pump inhibitors (PPI) for 2 weeks, prior to *H. pylori* detection (except for cases using serology for *H. pylori* detection);
(4) Alcohol or drug abuse;
(5) Decompensation of chronic diseases (cardiovascular, respiratory, gastrointestinal, endocrine diseases, and liver and kidney failure);
(6) Infectious and parasitic diseases, including human immunodeficiency virus (HIV), viral hepatitis, and tuberculosis;
(7) Diarrhea (bowel movements more than 3 times a day) for at least 3 consecutive days during the last month;
(8) Pregnancy or breastfeeding;
(9) A history of first-line eradication therapy;
(10) Inability or unwillingness to comply with the study procedures.

A total of 22 *H. pylori*-positive eligible patients aged 19 to 64 years were identified and enrolled in the study.

The following indications for *H. pylori* eradication in the enrolled *H. pylori*-positive patients were identified: duodenal ulcer disease (n = 4, 18.2%), non-investigated dyspepsia (n = 7, 31.8%), suspected long-term PPI use (n = 5, 22.7%), and atrophic gastritis confirmed via histology (n = 4, 18.2%). In 2 patients (9.1%), *H. pylori* was eradicated as a gastric cancer prevention measure.

The subjects were randomized into two treatment groups, as follows:

(1) ECAB-14 (n = 11): esomeprazole 20 mg, clarithromycin 500 mg, amoxicillin 1000 mg, and bismuthate tripotassium dicitrate 240 mg, twice daily, per os, for 14 days;
(2) ECAB-Z-14 (n = 11): esomeprazole 20 mg, clarithromycin 500 mg, amoxicillin 1000 mg, bismuthate tripotassium dicitrate 240 mg, twice daily, and butyric acid+inulin (Zacofalk) taken in 2 tablets (one tablet of butyric acid+inulin corresponds to 250 mg of butyric acid and 250 mg of inulin), daily, per os, for 14 days;

Fecal samples were collected from each subject at three time points, as follows:

(1) Prior to the beginning of eradication therapy to evaluate the baseline composition of the gut microbiota (time point I);
(2) After the end of eradication therapy (within 14 (+3) days from the beginning of eradication therapy) to evaluate the influence of the eradication therapy on the gut microbiota (time point II);
(3) Within a month (+5 days) of the end of eradication therapy to evaluate the long-term effects of *H. pylori* eradication on the gut microbiota (time point III).

The final study population consisted of 22 *H. pylori*-positive patients who passed the proposed *H. pylori* eradication therapy according to the allocated group. The stool samples of these patients were included for further analysis. No refusal or loss of follow-up were reported in the presented study.

Fecal samples were collected in disposable plastic containers. Samples were frozen on the same day and then stored at −80 °C until metagenomic analysis was performed.

### 2.2. Metagenomic Sequencing and Bioinformatic Analysis

The total DNA from the fecal samples was isolated using a FastDNA Spin Kit for Feces (MP Biomedicals, Santa Ana, CA, USA). Fragment libraries were prepared using an NEBNext Ultra II DNA Library Prep kit for Illumina (NEB, Ipswitch, MA, USA). The quality assessment was carried out using a 2100 Bioanalyzer system (Agilent Technologies, Santa Clara, CA, USA). A nucleotide sequence of the DNA library was determined with shotgun sequencing using a NextSeq 500 and HiSeq 1000 (Illumina, San Diego, CA, USA). Using the FastQC program, genomic data were preliminarily processed to include a selection of high quality reads, filter low quality reads, and correct reads using the method in [47]. Reads were mapped to the human reference genome, UCSC hg19, using the Bowtie 2 program [48]. Reads unmapped to the human genome were analyzed with the MetaPhlAn version 4.0 software package [49] to determine the taxonomic diversity of the community.

The qualitative and quantitative composition of the gut microbiota was assessed by studying the species and phyla of the microorganisms. The biodiversity index (the Shannon index) was used to assess the alpha diversity of the gut community. The Bray–Curtis distance was used to estimate the beta diversity of the gut microbiome.

Functional profiling was carried out for non-human reads using the HUMAnN3 tool [50] and MetaCyc database. Antibiotic resistance gene profiling was carried out using the Bowtie 2 [48] and feature Counts [51] tools and the CARD v. 3.2.5 database [52].

### 2.3. Statistical Analysis

We used a Wilcoxon signed rank test with Benjamini–Hochberg correction for multiple comparisons to assess differences between the alpha and beta diversity, and taxonomic and functional composition of the gut microbiota between time points in the same treatment group. *p* < 0.05 was considered significant.

## 3. Results

### 3.1. Diversity and Compositional Analysis

**Figure 1:**
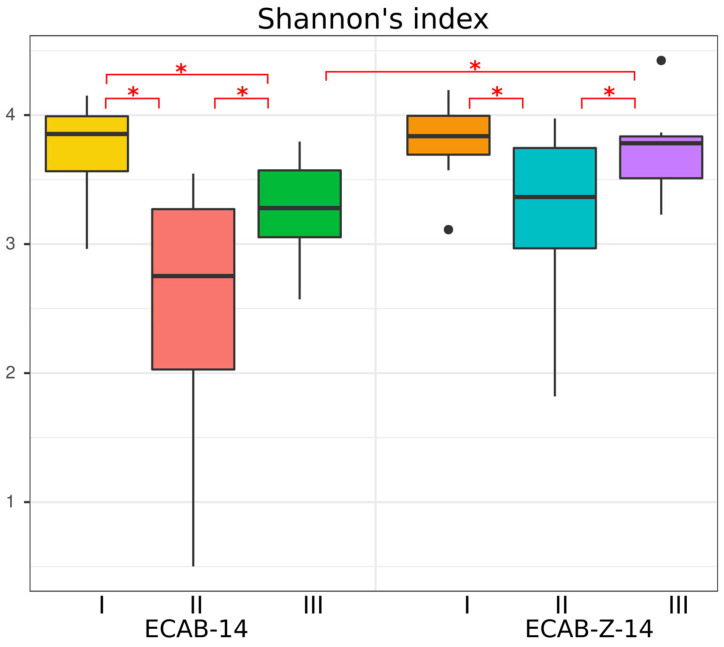
Alpha diversity estimated using Shannon indices between the ECAB-14 and ECAB-Z-14 *H. pylori* eradication treatment groups, *—*p* < 0.05 (Wilcoxon signed rank test for the same treatment groups and Wilcoxon rank sum test for the different treatment groups). Figure 1 shows boxplots with the median and first- and third-quartile values.

The Shannon index was calculated to evaluate the effects of *H. pylori* eradication therapy on the gut microbial community’s alpha diversity in both groups (Figure 1). Gut microbiota alpha diversity (the Shannon index) significantly decreased immediately after eradication therapy in both *H. pylori* eradication treatment groups (ECAB-14 and ECAB-Z-14) compared with the baseline, as follows: (2.46 ± 1.06) vs. (3.75 ± 0.342); *p* = 0.0029 and (3.22 ± 0.73) vs. (3.82 ± 0.30), *p* = 0.037 (the values in parentheses here and below represent the mean value ± standard deviation).

In the ECAB-Z-14 treatment group, the Shannon index increased and reached the baseline level one month after the end of therapy; no statistically significant differences were identified between time points I and III ((3.82 ± 0.30) vs. (3.71 ± 0.33), *p* = 0.206). However, for the ECAB-14 treatment arm, a reduction in the Shannon index was observed even up to a month after the end of *H. pylori* eradication therapy, as follows: (3.27 ± 0.39) vs. (3.75 ± 0.342), *p* = 0.019 (Figure 1). A reduction in the Shannon index might indicate an unstable state and reduced gut microbiota diversity, as well as the possible prevalence of one or more species.

**Figure 2:**
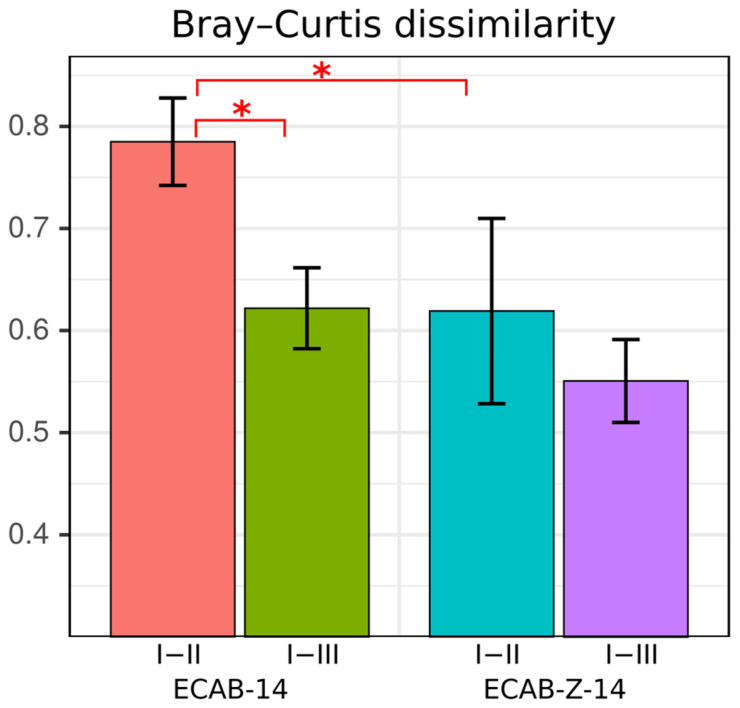
Bray–Curtis distance between ECAB-14 and ECAB-Z-14. *H. pylori* eradication treatment groups, *—*p* < 0.05 (Wilcoxon signed rank test for the same treatment group and Wilcoxon rank sum test for different treatment groups). Data are presented as the mean ± standard error of the mean.

PCoA was performed to evaluate alterations in community composition, as measured by Bray–Curtis metrics, which were calculated between time points I and II and I and III for each patient in the two comparison groups. A Bray–Curtis distance = 0 was considered to reflect exactly the same microbiota, and a Bray–Curtis distance = 1 was considered to indicate completely mismatched microbiota. Based on these results, it was found that the Bray–Curtis distance between points one I and II was significantly higher among patients on the ECAB-14 treatment regime than that among patients on ECAB-Z-14 eradication therapy (*p* < 0.05) (Figure 2). For this reason, we hypothesized that weaker deviations from the initial gut microbiota state could occur due to butyric acid+inulin supplementation in the eradication therapy regimen. There was also a significant difference in the Bray–Curtis distance between time points I and II, and I and III, among patients on the ECAB-14 therapy.

### 3.2. Analysis of the Taxonomic Composition of the Gut Microbiota

**Figure 3:**
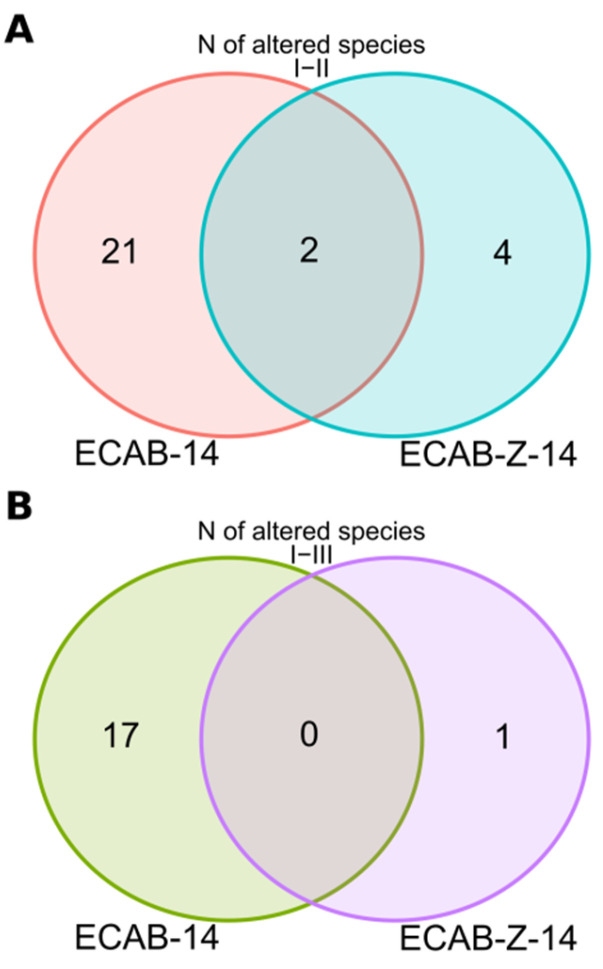
Venn diagram showing the number of bacterial species with altered levels of prevalence, *p* < 0.05. (**A**) Differences between time points I and II; (**B**) differences between time points I and III.

**Figure 4:**
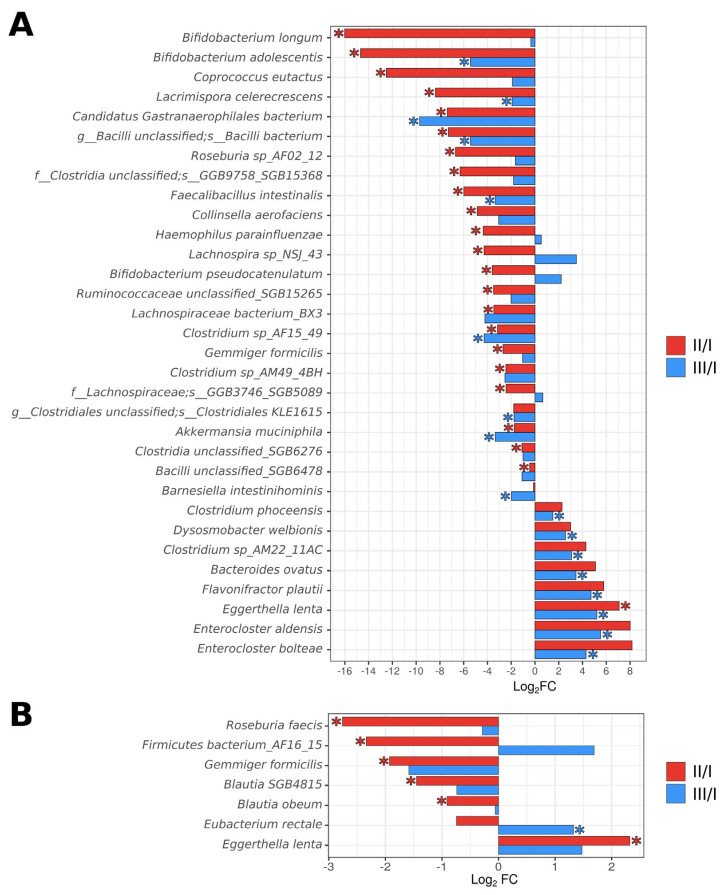
Bacterial species significantly differed between comparison groups. (**A**) ECAB-14; (**B**) ECAB-Z-14. Data are presented as the Log2 fold change in average relative abundance. Asterisks denote statistical significance determined by a Wilcoxon signed rank test with BH adjustment (*p* < 0.05).

Immediately after completing ECAB-Z-14 eradication therapy, we observed a statistically significant reduction in the relative abundance of the *Actinobacteria phylum* (time point II)—(4.16 ± 5.71)% vs. (6.93 ± 5.89)% before treatment, *p* = 0.037. At the same time, the abundance of six bacterial species (*Eggerthella lenta*, *Enterococcus faecium*, *Blautia SGB4815*, *Roseburia faecis*, *Gemmiger formicilis*, and *Firmicutes bacterium_AF16_15*) changed at time point II, compared with the baseline. In general, 4 weeks after the completion of therapy, the gut showed a tendency to return to its original microbial composition. However, the abundance of one bacterial species (*Eubacterium rectale*) significantly increased at time point III, compared with its initial level (Figure 3). Notably, among subjects who received the ECAB-14 treatment regimen, deeper changes were detected immediately after eradication therapy (time point II), and 4 weeks after the completion of eradication therapy (time point III), compared with the changes observed in the ECAB-Z-14 group. The abundance of 23 bacterial species significantly changed (decreasing in most cases) immediately after eradication therapy. In this treatment group, a positive trend was observed 4 weeks after the end of treatment, indicating the restoration of the original microbial composition. Nevertheless, the abundance of 17 bacterial species remained significantly altered (Figure 3 and Figure 4, Supplementary Table S1).

### 3.3. Functional Analysis

We also explored the alterations in the abundance of genes responsible for microbiota functional potential, possibly caused by *H. pylori* eradication therapy. Severe alterations in gene abundance, and hence, the gut microbiota’s functional potential, were found to be caused by *H. pylori* eradication therapy in both treatment arms, as follows: 112 signaling pathways differed significantly between time points I and II in patients receiving ECAB-14 therapy, whereas only 72 metabolic pathways (MP) were changed immediately after eradication therapy in the ECAB-Z-14 arm (Supplementary Table S2). Remarkably, 31 altered signaling pathways were found to be the same in both therapy regimes (Figure 4). Notably, the abundance of genes associated with the central pathway involved in the Fermentation to Butanoate process (CENTFERM-PWY: pyruvate fermentation to butanoate) was affected only in the ECAB-14 treatment arm (i.e., without adding butyric acid+inulin to the *H. pylori* eradication therapy).

**Figure 5:**
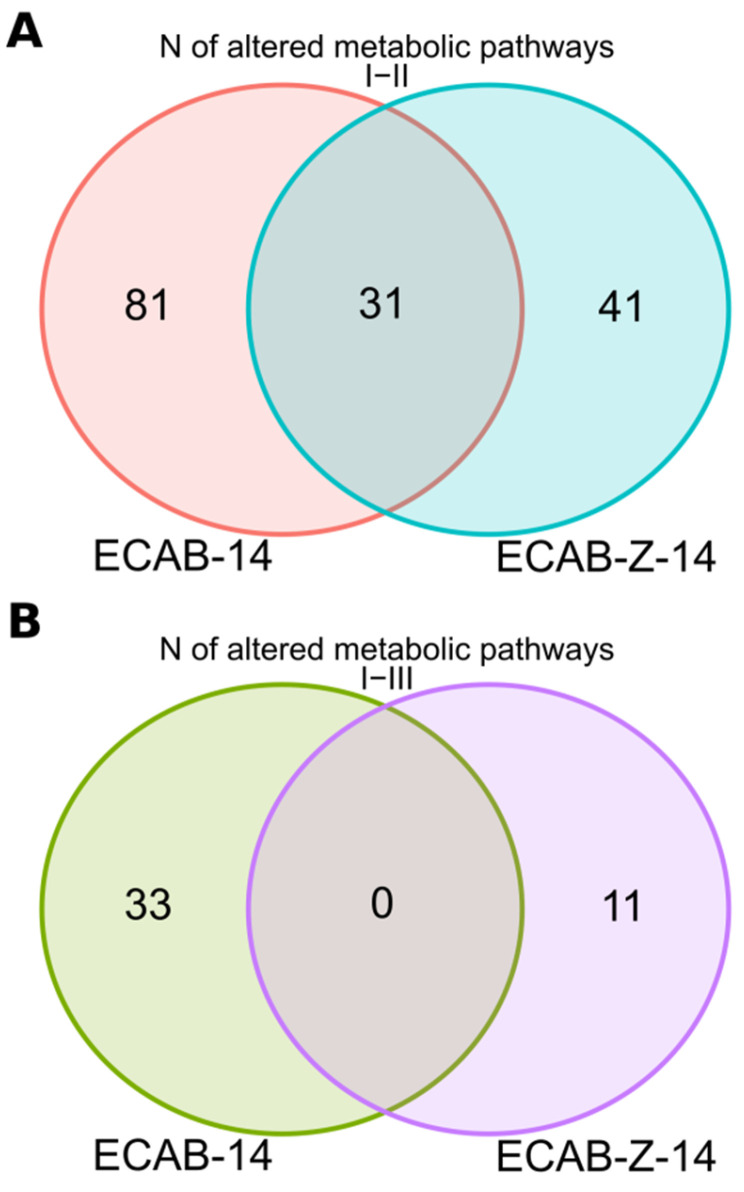
Venn diagram showing the number of genes responsible for microbiota’s functional potential with altered prevalence, *p* < 0.05. (**A**) Differences between time points I and II; (**B**) differences between time points I and III.

By the end of one month after eradication therapy, most changes in gut microbiota functional potential observed immediately after eradication therapy showed a tendency to return to their initial levels. However, changes in the abundance of 33 MP and 11 MP in the ECAB-14 and ECAB-Z-14 treatment arms, respectively, were still present (*p* < 0.05, Figure 5).

### 3.4. Gut Resistome Analysis

**Figure 6:**
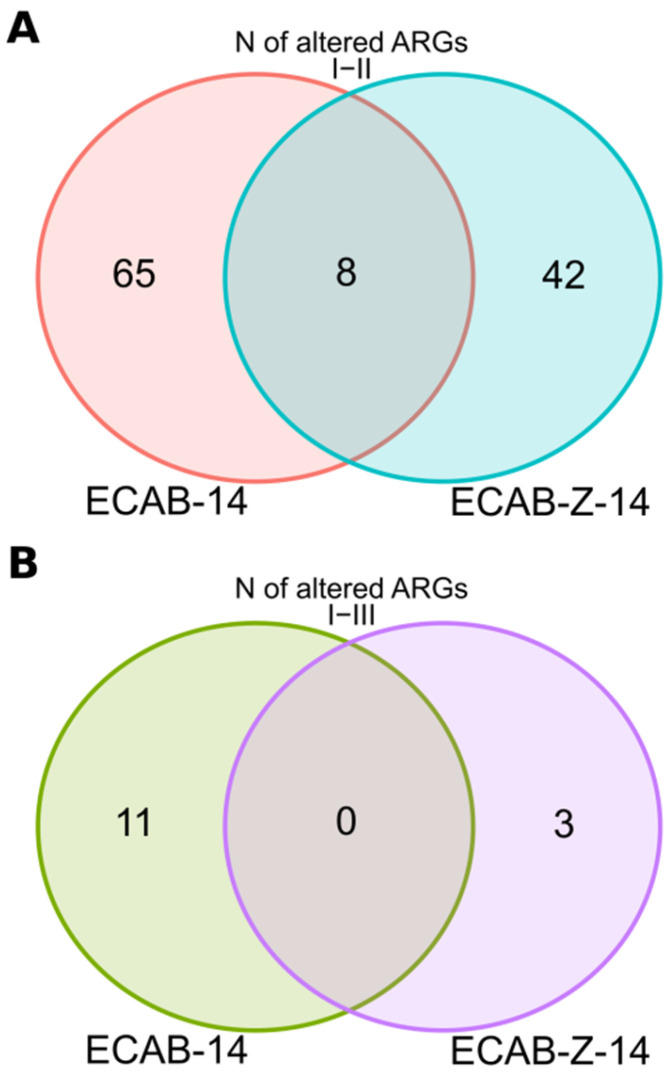
Venn diagram showing the prevalence of gut microbiota antibiotic resistance genes (ARG), *p* < 0.05. (**A**) Differences between time points I and II; (**B**) differences between time points I and III.

**Figure 7:**
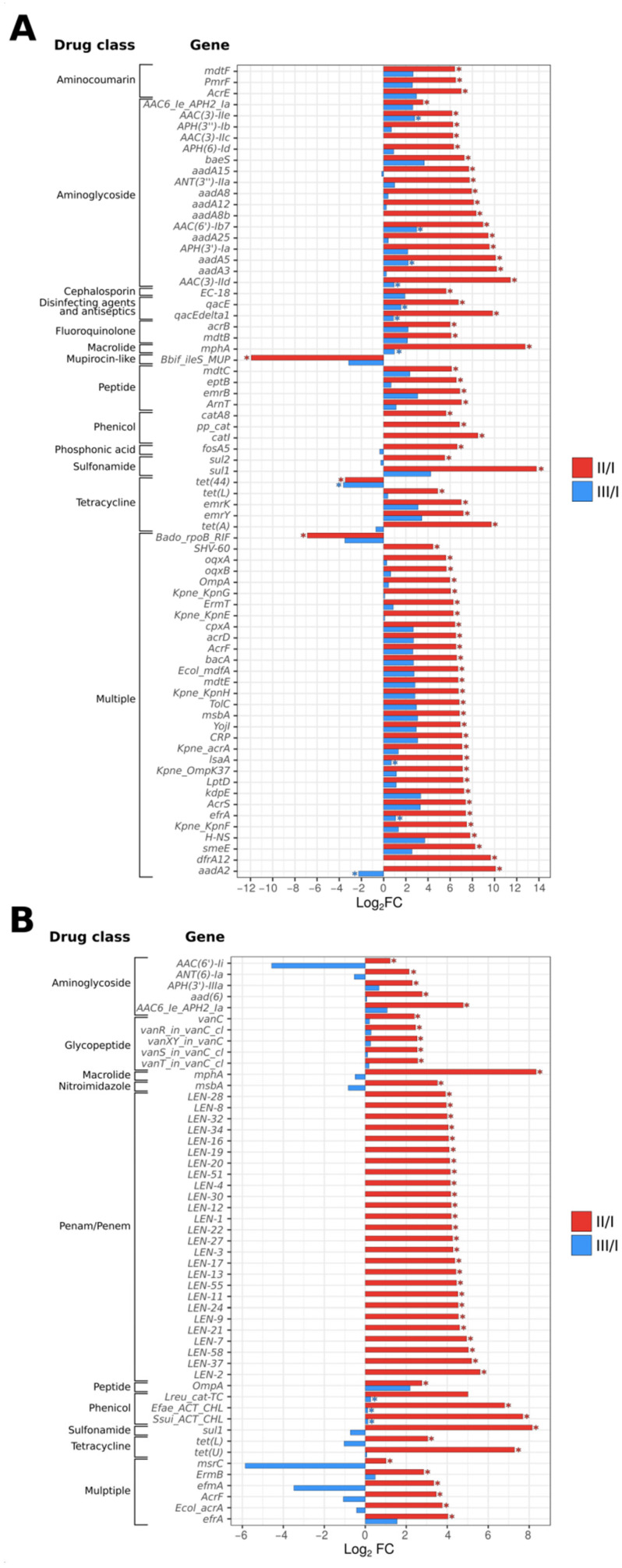
Antibiotic resistant genes significantly differed between comparison groups. (**A**) ECAB-14; (**B**) ECAB-Z-14. Data are presented as the Log2 fold change in average abundance (CPM + 1). Asterisks denote statistical significance determined by a Wilcoxon signed rank test with BH adjustment (*p* < 0.05).

*H. pylori* eradication therapy leads to substantial alterations in the gut resistome. A significant increase in the number of antibiotic resistance genes was observed in both treatment groups by the end of treatment, with more substantial and severe changes observed in the ECAB-14 treatment group. The abundance of 73 antibiotic resistance gut microbiota genes significantly increased immediately after completion of the eradication therapy. These genes were mainly related to β-lactam, aminoglycoside, fluoroquinolone, macrolide, and glycopeptide antibiotics. At the same time, the abundance of only 50 resistance gut microbiota genes, compared with the same antibacterial groups, increased when eradication therapy was supplemented with butyric acid+inulin (*p* < 0.05, Figure 6 and Figure 7, Supplementary Table S3).

It should be noted that eradication therapy results in the accumulation of genes that not only confer resistance to the antibiotics included in the eradication regimen, but also to several other groups of antimicrobial agents.

A tendency was observed, where the number of resistant genes decreased compared with the initial state. This occurred for most of the antibiotic resistant genes, 4 weeks after the end of therapy, in both treatment arms. However, a more positive trend was observed among patients treated with butyric acid+inulin-supplemented *H. pylori* eradication therapy, among whom, only three antibiotic resistance genes associated with phenicols remained unchanged. At the same time, the abundance of 11 antibiotic resistance genes still increased compared with the baseline level, even a month after the end of therapy, among patients receiving ECAB-14 eradication therapy (*p* < 0.05, Figure 6). These genes were mainly associated with resistance to aminoglycosides and macrolides, as well as multiresistance, caused by the efflux pumping mechanism.

## 4. Discussion

Several publications have described the detrimental impact of *H. pylori* eradication therapy on the composition of the gut microbiota using genome sequencing methods. Most relevant studies reported short- and long-term changes in gut microbiota composition after *H. pylori* eradication therapy, usually standard triple or bismuth quadruple therapy [10,11,12,13,14].

In general, all these studies showed the most pronounced changes in the composition of gut microbiota immediately after the completion of treatment, mostly due to a reduction in the abundance of “normal” representatives of gut microbiota, and an increase in the relative number of conditionally pathogenic bacteria, along with a reduction in the alpha diversity index. The predominant changes in most of the studies concerned a reduction in *Actinobacteria* populations (compared with baseline levels), an increase in *Proteobacteria* populations immediately after *H. pylori* eradication, and a return to baseline levels during the follow-up period. Unfortunately, some changes persisted up to a month after the end of treatment, which requires further investigation and may be of clinical importance [10,11,12,13,14]. However, some inconsistencies in the present results were observed, which could be explained by different study designs and national differences between the study populations, the regimens of eradication therapy used, the methodology used for evaluation, and the various time points used for gut microbiota assessments. Our study has some distinctive features. We used butyric acid+inulin adjuvant to *H. pylori* eradication therapy, which was expected to have both direct and indirect positive effects on the human gut microbiota. Although probiotics have been widely used and investigated as *H. pylori* eradication therapy supplements in various studies, data concerning the potential impact of butyric acid+inulin on the gut microbiota’s taxonomic composition and functional potential, particularly the gut resistome, remain limited.

Several studies have shown that supplementation with probiotics could improve the eradication rate and reduce the side effects caused by antibiotics. Antibiotic-induced changes in the gut microbiota may lead to diarrhea, abdominal bloating, and other side effects, which can be avoided through supplementation with probiotics [19,20,21]. Based on available data, in most cases, supplementation with probiotics was shown to diminish changes in the diversity and gut microbiota composition, compared with the control groups, without adding probiotic therapy [16,22,23,24,25,26]. Some studies have noted the benefits of probiotic supplementation, such as regulating the host isoflavone, fat, and energy metabolism, and reducing the risk of developing digestive disorders, gastrointestinal inflammation, and colorectal carcinoma [53]. However, the same authors reported the possibility of developing Parkinson’s disease and the depletion of key butyrate-producing bacteria after receiving multi-strain probiotic supplements. Therefore, although investigators have generally observed beneficial effects of probiotic treatment, the safety profile of probiotic supplementation remains unclear [27]. Moreover, it was recently reported that the exposing neonates to probiotics may be linked with a higher risk of oral, respiratory, and gastrointestinal infection [54].

The role of butyric acid and inulin in gut microbiota disorders caused by antibiotics remains unclear. Given the strong evidence that short-chain fatty acids (SCFAs) play a crucial role in colonocytes and the gut microbiota, we hypothesized that SCFA supplements, especially those, containing butyric acid (the most well-studied variety), may help mitigate the negative alterations caused by antibacterial therapy. We showed that the negative consequences of antibiotic-induced microbiota alterations, such as loss of species diversity, disruption in the number of bacterial species, and functional potential, were less-pronounced in the ECAB-Z-14 group (using supplements with butyric acid+inulin). The mechanisms underlying the positive impacts of butyric acid+inulin supplements on the gut microbiota remain unclear. Butyrate itself cannot be detected in the peripheral blood, indicating its fast metabolism in the colonic wall and/or liver. Located entirely in the digestive tract, butyrate is considered to act directly (inducing cell proliferation and renewal in necrotic areas) or indirectly (involving the hormono–neuro–immuno system) on tissue development and repair. Butyrate was also implicated in the down-regulation of bacteria virulence, likely through its direct effects on virulence gene expression and/or by acting on the cell proliferation of the host cells [55].

Butyrate stimulates beneficial flora and inhibits pathogen growth. These pathogens include Proteobacteria, which mainly increased after eradication therapy. Thus, in the ECAB-Z-14 group, it would be more feasible to observe less-pronounced changes in the diversity and abundance of bacterial species, and potentially achieve faster and smoother microbiota recovery to the baseline state, than in the ECAB-14 group. The other beneficial effect of butyrate is related to a reduction in the *Firmicutes*/*Bacteroidetes* ratio and the levels of several bacteria associated with a pro-inflammatory state while increasing the abundance of probiotic *Bifidobacterium* species [56,57]. It is known that inulin can reduce the abundance of *Bacteroidetes* and increase levels of *Bifidobacterium* spp., *Anaerostipes* spp., *Enterococcus faecalis*, and *Lactobacillus* spp. Inulin is known to promote an increase in the abundance of the genera *Phascolarctobacterium*, *Blautia*, *Akkermansia*, and *Ruminococcus*, as well as the *Lachnospiraceae* family, which are also responsible for SCFA production [58]. In our study, the same data were obtained. Compared with the initial point, the abundance of the genera *Bifidobacterium*, *Clostridium*, and *Lachnospira*, as well as the species *Akkermansia muciniphila*, among others, reduced in the ECAB-14 group immediately after therapy (point II). At the same time, the abundance of these bacteria was stable among patients from the ECAB-Z-14 group. These data support the protective role of butyrate and inulin. Compared with the initial point, 17 total species differed at time point III in the ECAB-14 group. *Eubacterium rectale*, which is able to degrade food-derived inulin to produce SCFAs [59], was the only species that significantly increased in the ECAB-Z-14 group compared with the initial point. Additionally, this bacterium produces endotoxin, which regulates the NF-kB immune response in normal colonocytes [60]. Thus, elevated levels of *E. rectale* can be considered potential markers of improved health.

There are limited data in the literature concerning the effects of *H. pylori* eradication therapy on the functional potential of the gut microbiota using whole-genome sequencing methods. For example, Oh B. et al. (2016) studied the impacts of probiotic supplementation (Medilac-S; *Streptococcus fecium* 9 × 108, *Bacillus subtilis* 1 × 108) on the structure and functional changes of the gut microbiota after *H. pylori* eradication (clarithromycin 500 mg, amoxicillin 1000 mg, and lansoprazole 30 mg twice a day, for 14 days) using whole-metagenomic sequence analysis [16]. In total, six patients aged 44 to 55 years were included in the study; the control group (*H. pylori* eradication without a probiotic supplementation) contained three patients. The relative abundance of functional genes differed between the triple eradication therapy and probiotic supplement groups after therapy [16].

Notably, we found that exposure to the *H. pylori* eradication therapy affected the functional potential of gut microbiota in both studied groups. However, the most pronounced changes were observed during conventional eradication therapy (ECAB-14) immediately after the end of treatment, with a tendency to return to initial levels one month after eradication therapy. Moreover, the abundance of genes responsible for the metabolic pathway associated with butyrate production (CENTFERM-PWY: pyruvate fermentation to butanoate) decreased only in the ECAB-14 treatment group. This result suggests that butyric acid+inulin supplements could minimize the potentially hazardous effects of antibiotics on the gut microbiome.

Although several studies have investigated the negative impacts of antibiotic exposure on the abundance of antibiotic resistant genes in the human gut, there are limited data that concern how *H. pylori* eradication influences the presence and prevalence of antibiotic resistant genes in the gut. The results of a study by Wang L. et al. (2022) observed dynamic alterations in the gut microbiota and abundance of antibiotic resistant genes induced by different regimens of eradication therapy (clarithromycin-based triple therapy, levofloxacin-based quadruple therapy, etc.) [15]. The most pronounced changes were observed in levofloxacin-containing quadruple therapy, which led to alterations of the tetracycline, macrolide-lincosamide-streptogramin, beta-lactam, aminoglycoside, and multidrug resistant antibiotic resistance gene classes. Compared with the baseline, the relative abundance of fluoroquinolone and multidrug and trimethoprim resistant genes significantly increased at 6 weeks and were restored 6 months after eradication [15]. Furthermore, certain microbial resistome profiles, such as *ermB*, which confers macrolide and *tetQ* gene resistance to tetracycline, were enhanced after *H. pylori* eradication therapy [1,61].

The results of our study generally align with those in the above-mentioned literature. The prevalence of antibiotic resistant genes in the gut microbiota increased significantly in both treatment groups by the end of the treatment. However, more severe alterations were observed in the ECAB-14 treatment group using the associated genes, with resistance to β-lactams, aminoglycosides, fluoroquinolones, macrolides, and glycopeptides found to be the most prevalent.

In the case of *H. pylori* eradication therapy supplemented with butyric acid+inulin, compared with ECAB-14 eradication therapy, we observed less-pronounced changes in the taxonomic composition, functional potential of the gut microbiota, and gut resistome immediately after, and one month after completion of eradication treatment. A trend of returning to the baseline state was noted during the follow-up period. This phenomenon was more positive and noticeable in the case of treatments adjusted with the butyric acid+inulin supplement (ECAB-Z-14 treatment group). Thus, the combination of prebiotic (inulin) and incapsulated metabiotic (butyric acid) can serve as an effective alternative to conventional probiotics due to the ability of this combination to recover homeostasis of the intestinal microbiota after antibiotic intake.

## Data Availability

The original contributions presented in the study are included in the article/Supplementary Materials, further inquiries can be directed to the corresponding author. Raw reads in the fastq format were deposited in the NCBI SRA under accession number PRJNA1063508 (https://www.ncbi.nlm.nih.gov/bioproject/1063508, accessed on 15 January 2024).

## Supplementary Materials

The following supporting information can be downloaded at: https://www.mdpi.com/article/10.3390/microorganisms12020319/s1, Table S1: Taxonomy; Table S2: Functions; Table S3: ABR_genes.
